# Creatine Kinase and Progression Rate in Amyotrophic Lateral Sclerosis

**DOI:** 10.3390/cells9051174

**Published:** 2020-05-08

**Authors:** Marco Ceccanti, Valeria Pozzilli, Chiara Cambieri, Laura Libonati, Emanuela Onesti, Vittorio Frasca, Ilenia Fiorini, Antonio Petrucci, Matteo Garibaldi, Eleonora Palma, Caterina Bendotti, Paola Fabbrizio, Maria Chiara Trolese, Giovanni Nardo, Maurizio Inghilleri

**Affiliations:** 1 Rare Neuromuscular Diseases Centre, Department of Human Neurosciences, Sapienza University of Rome, 00185 Rome, Italy; marco.ceccanti@yahoo.it (M.C.); valeria.pozzilli@yahoo.co.uk (V.P.); chiara.cambieri@gmail.com (C.C.); laura.libonati@yahoo.it (L.L.); emanuela.onesti@uniroma1.it (E.O.); vittorio.frasca@uniroma1.it (V.F.); ilenia.fiorini77@libero.it (I.F.); 2 Centre for Neuromuscular and Neurological Rare Diseases, San Camillo Forlanini Hospital, 00152 Rome, Italy; anpetrucci@scamilloforlanini.rm.it; 3 Neuromuscular Disease Centre, Department of Neurology, Mental Health and Sensory Organs (NESMOS), Sant’Andrea Hospital, Sapienza University of Rome, 00189 Rome, Italy; matteo.garibaldi@uniroma1.it; 4 Department of Physiology and Pharmacology, Laboratory Affiliated to Istituto Pasteur Italia, Sapienza University of Rome, 00185 Rome, Italy; eleonora.palma@uniroma1.it; 5 Laboratory Molecular Neurobiology, Department of Neuroscience, Istituto di Ricerche Famacologiche Mario Negri-IRCCS, 20156 Milan, Italy; caterina.bendotti@marionegri.it (C.B.); paola.fabbrizio@marionegri.it (P.F.); mariachiara.trolese@marionegri.it (M.C.T.); giovanni.nardo@marionegri.it (G.N.)

**Keywords:** prognosis, amyotrophic lateral sclerosis, chronic inflammatory demyelinating polyneuropathy, CK, creatine kinase, neurodegenerative disease

## Abstract

Amyotrophic lateral sclerosis (ALS) is a neurodegenerative disease with no recognized clinical prognostic factor. Creatinine kinase (CK) increase in these patients is already described with conflicting results on prognosis and survival. In 126 ALS patients who were fast or slow disease progressors, CK levels were assayed for 16 months every 4 months in an observational case-control cohort study with prospective data collection conducted in Italy. CK was also measured at baseline in 88 CIDP patients with secondary axonal damage and in two mouse strains (129SvHSD and C57-BL) carrying the same SOD1G93A transgene expression but showing a fast (129Sv-SOD1G93A) and slow (C57-SOD1G93A) ALS progression rate. Higher CK was found in ALS slow progressors compared to fast progressors in T1, T2, T3, and T4, with a correlation with Revised Amyotrophic Lateral Sclerosis Functional Rating Scale (ALSFRS-R) scores. Higher CK was found in spinal compared to bulbar-onset patients. Transgenic and non-transgenic C57BL mice showed higher CK levels compared to 129SvHSD strain. At baseline mean CK was higher in ALS compared to CIDP. CK can predict the disease progression, with slow progressors associated with higher levels and fast progressors to lower levels, in both ALS patients and mice. CK is higher in ALS patients compared to patients with CIDP with secondary axonal damage; the higher levels of CK in slow progressors patients, but also in C57BL transgenic and non-transgenic mice designs CK as a predisposing factor for disease rate progression.

## 1. Introduction

Amyotrophic lateral sclerosis (ALS) is a neurodegenerative disease involving motor neurons of the motor cortex, the brainstem, and the spinal cord, with two possible onset phenotypes: spinal or bulbar onset. Diagnosis can be made with clinical and electrophysiological examination; neuroimaging and laboratory analysis can exclude other diseases [1,2]. Some techniques can demonstrate upper motor neuron [3,4] and extra motor [5,6,7,8] involvement. No serum biomarker is approved for monitoring or forecast disease evolution [9].

Chronic inflammatory demyelinating polyradiculoneuropathy (CIDP) is an autoimmune disorder that causes demyelination and subsequent axonal damage to nerves. Motor involvement occurs in up to 94% of patients [10].

A moderate elevation of creatinine kinase (CK) in subsets of ALS patients is already known [11,12,13,14,15,16,17,18,19,20,21,22,23]. Recently, there has been a closer look into CK levels over time to understand if they can predict prognosis and survival, but results are conflicting [20,21,22]. CK can catalyze the conversion of phosphocreatine and ADP to creatine and ATP, buffering energy for muscle contraction [24]. CK elevation is commonly due to injury of the sarcolemma [25]. The increased CK levels in ALS patients was associated with the motor neuron loss, denervation, and muscular atrophy [23], or to an up-regulation from the muscle to provide energy [21]. However, up to now, it is still unclear if the CK increases as a compensatory mechanism to up-regulate the muscular metabolic pathways or if it is already increased in ALS patients with different progression rate.

Another crucial molecule, the myoglobin, is a reservoir of oxygen during muscular exercise [26] and increases in myopathies and in vigorous exercise [27]. Immunohistochemical staining in ALS showed a preserved immunoreactivity for myoglobin, whilst dystrophies and polymyositis have a reduced or absent staining [28,29]. 

The aim of this study is to elucidate the utility of measuring CK to forecast the disease progression in ALS patients and to compare CK and Myoglobin (Mb) levels in ALS and in a cohort of CIDP patients affected by secondary axonal damage.

Despite the different etiology with ALS, the cohort of CIDP patients we included also had chronic denervation and a reduced motor neuronal pool, similar to ALS patients. The reduction of motor neurons increases the firing rate of the residual ones, as a compensatory mechanism. This could up-regulate the muscular metabolism and, consequently, the CK levels as it happens in the heavy muscular activity [30]. The CIDP cohort could settle if the CK increase is specific for ALS or an epiphenomenon of motor neuronal loss.

Like ALS patients, ALS mouse models, with the same gene mutation, show a significant variability in the disease severity due to their genetic background. We observed that transgenic SOD1G93A mice on C57BL/6JOlaHsd genetic background (C57-SOD1G93A), indicated as slow progressor, exhibited a delay in the onset of symptoms and a prolonged survival of about 3 and 8 weeks, respectively, compared to the SOD1G93A mice on 129SvHSD strain (129Sv-SOD1G93A), indicated as fast progressor despite they carry the same amount of transgene and express the same amount of mutant protein [31,32]. Such difference is independent of the spinal motor neuron soma loss, which is affected at the same extent in both strains, while a prominent role of the peripheral neuromuscular system seems responsible for the difference in the disease severity [33].

## 2. Materials and Methods

This is an observational case-control cohort study with prospective data collection, involving data from 126 Caucasian patients affected by clinically definite, probable or laboratory supported probable ALS, as defined by the revised El Escorial Criteria [1] and 88 patients affected by CIDP with secondary axonal damage demonstrated by EMG and motor NCS (EFNS/PNS criteria [34]).

Bulbar onset patients showed a classic ALS pattern, with muscle atrophy and neurogenic features at the time of recruitment. Patients were consecutively recruited from June 2017 to October 2019 in Sapienza University of Rome and San Camillo Forlanini Hospital. 

Exclusion criteria were the following: EMG testing or intramuscular injections within 10 days before the blood test, being under statins, isotretinoin, antiretrovirals, colchicine, neuroleptics, hydroxychloroquine, being affected by other diseases that can determine an elevation of CK and Mb i.e., dystrophies, myopathies, myositis, hyper/hypothyroidism, renal insufficiency.

At baseline, all ALS and CIDP patients underwent venous blood biochemistry tests, including CK and Mb, after overnight fasting and rest. CK assay was performed by direct enzymatic level according to the International Federation of Clinical Chemists with a normal range of 24–195 U/I for men and 24–170 for females. Given the similar normal range, no statistical sub-analysis for gender was performed. Mb levels were determined by radioimmunoassay with a normal range of 25–72 ng/mL.

At baseline, all ALS patients also underwent motor NCS bilaterally on the medial plantar nerve and ulnar nerve and relationship between cMAP results and CK levels at baseline was examined.

Furthermore, ALS patients were divided into fast or slow disease progressors according to monthly reduction in ALSFRS-R score from start of symptoms to baseline. A progression index (PI) was employed to differentiate patients based on their rate of disease advancement: PI = (48-ALSFRS-R score at baseline)/disease duration from onset of symptoms (months). Slow progressors had PI ≤0.5, fast progressors had PI > 0.5. In fact, a previous study demonstrated that patients with a monthly reduction of ALSFRS-R score lower than 0.5 had a better survival compared to patients with higher scores [35].

All ALS patients were followed-up for 16 months; CK was serially dosed every 4 months (T1–T5). Relationship between fast or slow rate of progression and CK levels in single time points was examined.

### 2.1. Mice

Female transgenic SOD1G93A mice on C57BL/6JOlaHsd or 129SvHSD genetic background, hereafter indicated as C57SOD1G93A and 129SvSOD1G93A, respectively, and corresponding non-transgenic (Ntg) littermates were used. The animals were housed under SPF (specific pathogen-free) standard conditions (22 ± 1 °C, 55 ± 10% relative humidity and 12-h light/dark schedule), 3–4 per cage, with free access to food (standard pellet, Altromin, MT, Rieper) and water.

The blood was collected from the submandibular plexus of anesthetized mice and centrifuged at 12,000 rpm for 10 min. The serum was separated and stored at −80 °C. Creatine kinase determination was performed by standard spectrophotometric analysis by using the Pointe Scientific Creatine Kinase (CK10) reagent (Fischer Scientific, Kalamazoo, MI, USA). Absorption at 340 nm was measured every minute for 3 min at 37 °C to calculate the enzymatic activity. Duplicate measurements were done on each serum sample. Creatine kinase activity is expressed in units per liter.

Blood was collected from both SOD1G93A mouse strains and respective non-transgenic littermates at 12 weeks of age before symptoms appearance and at the onset of muscle force deficit corresponding to 14 weeks and 18 weeks age for the 129Sv-SOD1G93A and the C57-SOD1G93A, respectively [31].

### 2.2. Standard Protocol Approvals, Registrations, and Patient Consents

This study was approved by the ethical committee of Policlinico Umberto I, Rome, for any experiments using human participants and written consent was obtained from all participants of the study, according to Declaration of Helsinki.

Procedures involving animals and their care were conducted in conformity with the institutional guidelines of the Mario Negri Institute for Pharmacological Research, Milan, Italy, which are in compliance with national (D.lgs 26/2014; Authorization n.19/2008-A issued March 6, 2008 by Ministry of Health) and Mario Negri Institutional regulations and Policies providing internal authorization for persons conducting animal experiments (Quality Management System certificate—UNI EN ISO 9001:2008—reg. No. 6121); the NIH Guide for the Care and Use of Laboratory Animals (2011 edition) and EU directives and guidelines (EEC Council Directive 2010/63/UE).

### 2.3. Aims of the Study

Primary endpoints of this study were to investigate CK values in fast and slow ALS progressors and in the different onset phenotypes; possible correlations with cMAP measures were also assessed. Finally, CK and Mb values were compared in ALS and CIDP with secondary axonal damage to evaluate differences in these diseases with different pathogenesis but with the same effects on the lower motor neurons.

### 2.4. Statistical Analysis

The statistical analysis was performed using SPSS software 25.0. Qualitative variables have been described with frequency distributions, while quantitative variables have been described with mean levels ± standard error mean (S.E.M.).

Mann-Whitney U test for independent samples was used to compare CK and Mb levels between ALS patients and CIDP patients at baseline.

Kruskal-Wallis test for independent samples has been used to compare CK levels in spinal vs. bulbar onset patients, and fast vs. slow progressive patients and to compare ALSFRS-R score and time since diagnosis in fast vs. slow progressive patients.

Pearson’s chi-squared test was used to compare bulbar/spinal onset and male/female prevalence in fast and slow progressive patients.

Pearson correlation coefficient was used to examine the relationship between CK and cMAP levels at baseline and relationship between CK and ALSFRS-R scores in each time point. ANOVA for repeated measures (RM-ANOVA) was employed to verify the effect of time in the single time points and the effect of rate of progression on single levels of CK.

A *p*-value of <0.05 was regarded as statistically significant.

## 3. Results

A global amount of 126 ALS patients (78 males and 48 females) were included, with a mean age of 66 ± 12.6 years (range 44–90). The mean period from start of symptoms to study entry was 26.3 months (median 17.4 months).

A total of 88 CIDP patients (53 males and 35 females) were recruited with a mean age of 65 ± 16.3 years (range 32–86).

Demographic characteristics of the sample are shown in Table 1.

### 3.1. Baseline CK and Mb in ALS Patients

Table 2 shows baseline CK and Mb levels in ALS (by site of onset and rate of progression). In ALS, spinal onset patients had mean CK levels higher than bulbar onset patients (*p* < 0.01). Myoglobin levels also were higher in spinal compared to bulbar onset patients (*p* = 0.032).

When subdivided according to rate of progression, at baseline slow progressive patients showed higher CK and Mb levels compared to fast progressive patients (*p* = 0.024).

Mean levels of cMAP in ALS patients from medial plantar nerve (8.1 mV ± 0.6) and ulnar nerve (7.4 ± 0.5 mV), both obtained from the mean of measurements from the right limb and the left limb, were under the normal range. No significant correlation with CK levels was found.

### 3.2. Relationship between CK and FAST/SLOW Progression over Time

**Figure 1:**
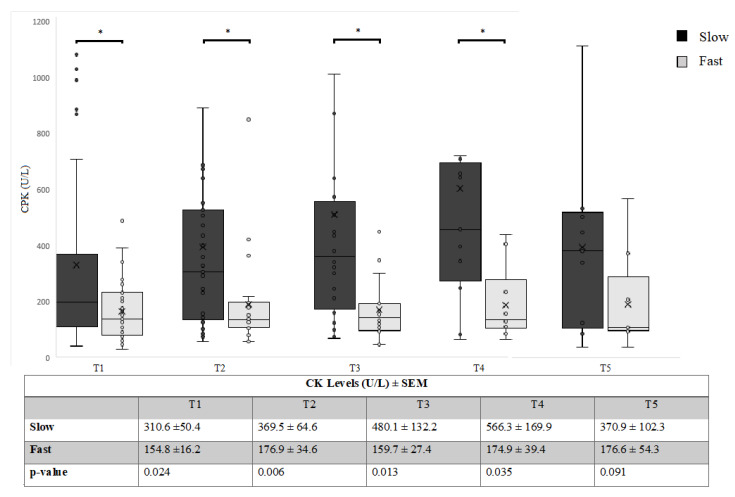
Mean CK levels (U/L) in slow vs. fast progressive ALS patients. * *p* < 0.05.

Figure 1 compares mean CK levels of slow and fast progressive patients during follow up in the single time points, showing significantly higher CK levels in slow progressors at T1, T2, T3, and T4, but not at T5; the power of the test was >80% for T1–T4 and <80% for T5.

**Figure 2:**
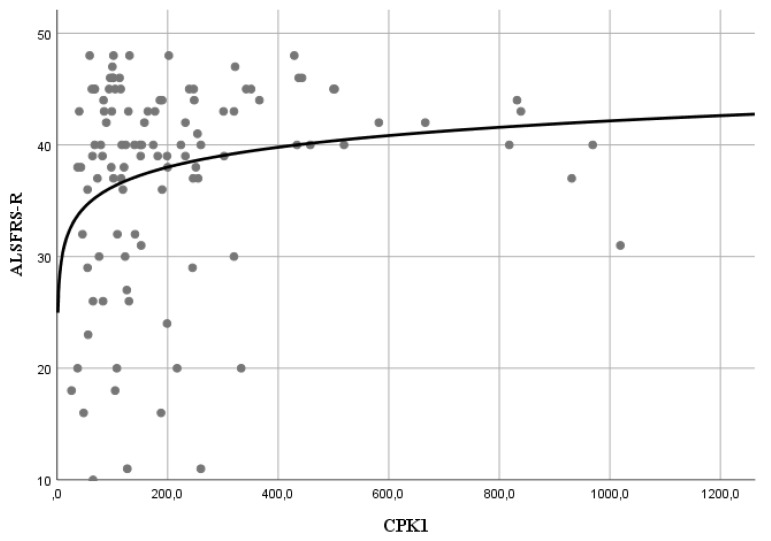
Correlation between ALSFRS-R score and CK levels at T1.

Moreover, a logarithmic correlation was demonstrated between CK values and ALSFRS-R score administered to patients in the single time points in T1 (Figure 2), T2, T3, and T4 but not in T5, where there was still a not significant correlation (Figure 2; Table 3).

A RM ANOVA with assumption of sphericity demonstrated a global within-subject effect of CK levels over time in the ALS patients [F (4.72) = 4.885, *p* = 0.002]. The between-subjects analysis showed a difference related to the rate of progression group (slow or fast) (*p* = 0.028).

### 3.3. CK and Mb in ALS and CIDP

**Figure 3:**
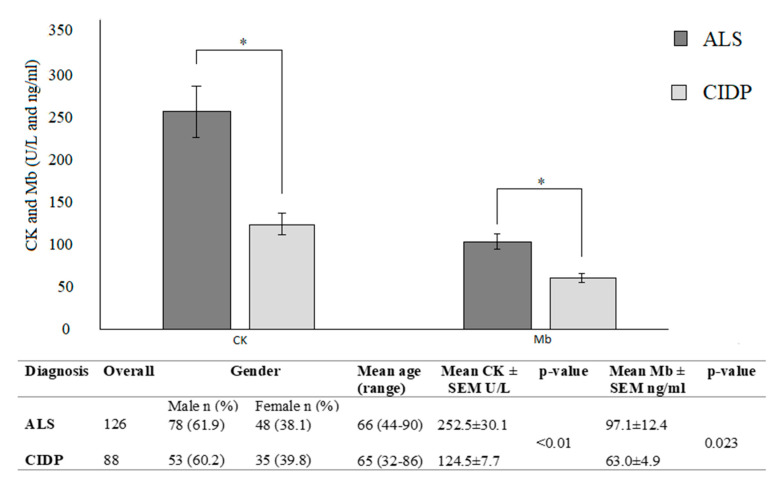
Mean CK and Mb in ALS patients vs. Chronic inflammatory demyelinating polyradiculoneuropathy (CIDP) patients. * *p* < 0.05.

Figure 3 shows comparison between CK and Mb in ALS and CIDP. At baseline, 47% of ALS patients had levels above the upper limit of normal range, and 3 patients had levels >1000 U/L. 14% of CIDP patients had levels above the upper limit of normal range at the baseline, but the higher registered level was 421 U/L. There was a significant difference between CK and Mb levels in ALS compared to CIDP.

### 3.4. CK in Fast and Slow-Progressive ALS Mouse Models

**Figure 4:**
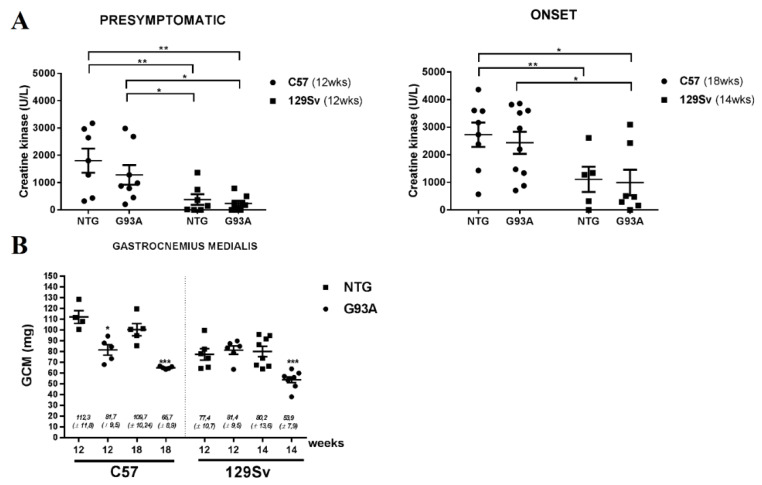
CK serum levels (U/L) in slow and fast progressive ALS mice. (**A**) CK levels were measured in mouse serum of C57-SOD1G93A (slow), 129Sv-SOD1G93A (fast), mice and relative controls (NTG) at pre-symptomatic (12 weeks) and onset (18 and 14weeks respectively) disease stages. CK levels were significantly higher in C57 mice than 129Sv mice, regardless of SOD1G93A mutation. Statistical significance was calculated by Two-Way ANOVA with Sidak’s post-analysis. Data are presented as mean ± SEM. * *p* < 0.05; ** *p* < 0.001. (**B**) Muscle wasting was calculated by measuring of the gastrocnemius medialis (GCM) muscle weight of C57-SOD1G93A and 129Sv-SOD1G93A mice and relative NTG littermates. Statistical significance was calculated by Mann and Whitney test. Data are presented as mean ± SEM. * *p* < 0.05; *** *p* < 0.0001.

When we examined the serum levels of CK in fast and slow progressors mice we found that CK levels progressively increased with the age of mice in both strains (129Sv vs. C57), independently of the genotype (NTG vs. SOD1G93A). However, the mouse strain associated with the slow progression of the disease (C57) at all time points showed significantly higher levels compared to the strain of fast progressing mice (129Sv) (Figure 4A). CK is produced for the major part in the skeletal muscle Interestingly, we found that the weight of gastrocnemius muscles of the C57 NTG mice is 55% higher than those of 129Sv mice at the same age (Figure 4B).

## 4. Discussion

CK and Mb are mainly present in striated skeletal muscle. CK is an enzyme composed of two isoforms, which can be either a B (brain) or M (muscle) type, giving rise to three isoenzymes: CK-MM, CK-BB and CK-MB. CK-MM is the predominant form in the muscle and catalyzes the phosphocreatine reaction causing production of ATP. Mb instead is a globular protein, found especially in type 1 slow-twitch muscle fibers, and acts as a deposit of oxygen, supporting diffusion of O_2_ from blood vessels to mitochondria during aerobic exercise. Notoriously, a rise of CK and Mb levels in serum is secondary to rhabdomyolysis, happening in myopathies, myositis, and in heavy muscular exercise [30]. In ALS, muscles are characterized by neurogenic atrophy due to denervation [35]. Muscle biopsies show neurogenic changes essentially in all ALS patients, while necrosis is seen in a very small percentage of them [36]. Consequently, raised serum levels of CK and Mb in ALS patients must be due to a mechanism other than the lysis of muscle fibers.

This study revealed that slow progressors ALS patients, i.e., patients with low monthly reduction in ALSFRS-R score, showed higher CK levels both at baseline and up to 12 months follow up. The latter also presented a constant increase in CK levels over time, whilst fast progressors showed low but fairly stable CK levels over time (Figure 1). CK increase over time in the slow progressive patients could be due to the earlier death of patients with lower CK, thus increasing the CK average in the group. The lack of differences in CK between the two groups at 16 months should be interpreted considering the reduction of the sample size, as demonstrated by the low power of the test.

CK levels also showed a logarithmic correlation with ALSFRS-R scores (Figure 2) at every single time point up to 12 months; this means that for low blood CK levels, minor variations in CK levels are associated with great variations in ALSFRS-R score.

Mb as well showed significantly higher levels in slow compared to fast progressors. However, our study considered only baseline measurements, which nevertheless were useful indicators for rate of advancement.

In this study, fast and slow progressive patients had similar ALSFRS-R score at baseline, being the time since diagnosis longer in slow than fast progressors. This enhances our results and reveals that independently from the functional scores at one time, fast progressors have lower CK blood levels compared to slow progressors.

The mouse model confirms this observation: C57BL and 129SvHSD mouse strain differ in terms of CK levels, with the strain related to the slow progressor mice (C57BL) showing higher CK compared to that of fast progressors (129svHSD) in both transgenic and non-transgenic littermates. Our data are consistent with a previous study showing that blood CK levels were approximately 6 fold higher in C57BL/6J than in 129Sv/HSD mice 2 months old although at the later ages (8–12 months) such difference disappeared and the CK levels decrease [37].

CK is an important enzyme for tissues that consume ATP rapidly, like the skeletal muscle, serving as an energy reservoir for the rapid buffering and regeneration of ATP in situ. Therefore, we hypothesize that C57 mouse strain express a higher metabolic reservoir, which preserves this strain from a rapid loss of strength in consequence of SOD1G93A mutation. Although we ignore whether the levels of CK or ATP in the skeletal muscle are different between the two mice strains, we previously found that 129Sv SOD1G93A had an impaired production of ATP in the spinal cord in comparison to the C57-SOD1G93A [38]. This may indicate that 129Sv mice are unable to maintain proper energy homeostasis in different compartment including the skeletal muscle possibly due to a lower expression of CK. In Figure 4 we show that muscle mass is higher in C57 than in 129Sv mice suggesting that such difference could explain the difference in serum CK levels. However, apparently, this correlation between muscle mass and serum CK levels does not comply with the fact that CK levels are maintained at high levels in C57SOD1G93A mice even in presence of a progressive reduction of muscle mass in these mice at 12 and 18 weeks age with respect to their NTG littermates. A similar phenomenon was observed in 129SvSOD1G93A mice of 14 weeks age compared to the respective NTG littermates. This suggests an increased CK production from the residual muscle fibers of both SOD1G93A mouse strain. However, since levels remain higher in the C57 than 129Sv mouse stains this can be interpreted as a metabolic predisposing factor in the C57 mice, favoring a better disease progression in mice with higher CK and metabolic reservoir. Thus, fast or slow progression rate must be associated with baseline CK and genetic background.

Indeed, different CK values were also described in different human genotypes: afroamericans are known to be strongly associated with high CK values, regardless of the gender, with 97.5th percentile of CK being 382 U/L in white US men and 1001 U/L in black US men [39,40]. Moreover, afroamericans have a lower incidence of ALS [41] and a longer disease duration, with the 75th percentile for survival of 53 months in African Americans and 40 months in whites [42,43]. Our data are consistent with all these reports.

A potential limit of the study is the higher prevalence of spinal onset in slow compared to fast progressor ALS patients. We highlight that the patients were classified in bulbar or spinal according to the site of onset, but at the time of the recruitment all the patients had a clinically definite, probable or laboratory supported probable ALS, as defined by the revised El Escorial Criteria, and all bulbar onset patients had an involvement of the upper or lower limbs, thus making the sample more homogeneous.

Recent researches [22,23] have considered that an increase in CK levels in ALS may be linked to the entity of denervation potentials. Even if a leakage of CK from muscle fibers can be hypothesized, also due to an increase in myoglobin levels, we highlight that denervation is a mechanism that induces nicotinic acetylcholine receptor spreading [44], but not necrosis [45] or increase in membrane permeability. Moreover, we found lower CK levels in fast progressors mice, which have more denervation than slow progressors (33).

Another study [21] hypothesized a link between blood CK and metabolic upregulation in the muscle of ALS patients, in order to increase energy production, demonstrating also a correlation between logCK and survival, and this correlation is confirmed by our findings. The authors hypothesize a CK up-regulation to provide energy to the muscle of ALS patients. The higher CK levels in C57BL than in 129SvHSD mouse strains suggests an increase in muscular metabolism as a mechanism compensative to the disease progression. We acknowledge that many genetic differences other than CK values exist between the C57BL and 129SvHSD genetic backgrounds; nevertheless, we highlight that the two strains express the same number of transgene and the same amount of mutant protein and that we are not attributing a pathogenic role to CK, but simply indicating it as a possible prognostic marker. Similar analyses could be performed on other mouse genetic background in the future.

Myoglobin has been studied less in ALS compared to CK; one study [46] demonstrated that in ALS overall there is a moderate increase in Mb levels. Another study [28] demonstrated that Mb immunoreactivity was preserved in muscle fibers with denervation atrophy, while marked decrease or loss of Mb occurs in muscle fibers characterized by necrosis and therefore definite disruption of the muscle fibers. No correlation has ever been made with the rate of progression or survival in ALS. Elevation of serum Mb levels in slow progressors, but not in fast progressors, at baseline can be interpreted as a protective factor.

All these findings about myonecrosis enzymes could confirm a central role of the muscle in the pathogenesis, prognosis, and possible therapeutic target of ALS [47,48,49].

Our study deepened the etiology of CK and Mb elevation in ALS by measuring CK and Mb in a control group of patients affected by CIDP with secondary axonal damage. We considered this particular control group as it shares with ALS a similar axonal damage and high frequency motoneuronal discharge of the remaining axons, thus supplying the damaged ones. The increased motor neuronal firing rate could up-regulate the muscular metabolism and, consequently, the CK levels. This study demonstrates a significant difference in both CK and Mb levels between the two groups, being higher in ALS compared to CIDP; hence we hypothesize that increase in serum levels of the two molecules cannot be due to the high frequency motoneuronal discharge and subsequent up-regulation of the muscular metabolism. We confirmed this hypothesis also by carrying out nerve conduction studies on ALS patients without any relationship between cMAP scores and CK levels. This data confirms that the increase of CK and Mb levels in ALS patients cannot be due a compensatory mechanism after motor neuron damage.

## 5. Conclusions

Serum CK and Mb can be a useful tool to predict and monitor ALS disease progression, as higher levels are linked to a slow progression of disease. This may be interpreted as a predisposing factor, with patients with higher muscular metabolic reservoir, which show a slower progression rate. This study shows that CK and Mb levels are not increased in other neuropathies with motor axonal damage, such as CIDP, suggesting a central role of the muscle as a possible therapeutic target in ALS.

## Figures and Tables

**Table 1:** Demographic characteristics of the sample.

	Fast	Slow	*p*-Value
Time since diagnosis (months ±SEM)	15.4 ± 2.2	34.6 ± 4.8	<0.05
ALSFRS-R (±SEM)	36.6 ± 1.7	38.26 ± 0.8	>0.05
Bulbar/spinal onset (n)	19/27	20/60	<0.05
Male/female (n)	26/22	47/31	>0.05

**Table 2:** Baseline creatinine kinase (CK) and Myoglobin (Mb) in amyotrophic lateral sclerosis (ALS) by site of onset and rate of progression.

		N	CK Levels (U/L) ± SEM	*p*-Value	Mb Levels (ng/mL) ± SEM	*p*-Value
**Overall**		126	252.5 ± 30.1		97.1 ± 12.4	
**Site of onset**	Spinal	91	287.2 ± 39.7	<0.01	104.1 ± 15.9	<0.05
	Bulbar	35	161.4 ± 29.9		78.9 ± 18.7	
**Rate of progression**	Slow	79	310.6 ± 50.4	<0.05	116.9 ± 15.8	<0.05
	Fast	47	154.8 ± 16.2		63.7 ± 10.3	

**Table 3:** CK and ALSFRS-R progression over time.

	T1	T2	T3	T4	T5
**N**	126	115	102	96	61
**CPK ± SEM**	252.5 ± 30.1	297.7 ± 44.3	360.6 ± 76.8	420.3 ± 88.1	298.3 ± 69.5
**ALSFRS-R ± SEM**	37.6 ± 0.87	35.5 ± 1.3	33.6 ± 1.5	31.9 ± 2.1	27.8 ± 2.8
